# Distance Error Correction in Time-of-Flight Cameras Using Asynchronous Integration Time

**DOI:** 10.3390/s20041156

**Published:** 2020-02-20

**Authors:** Eu-Tteum Baek, Hyung-Jeong Yang, Soo-Hyung Kim, Gueesang Lee, Hieyong Jeong

**Affiliations:** Department of Electronics and Computer Engineering, Chonnam National University, 77 Yongbong-ro, Gwangju 61186, Korea; geodo100@gmail.com (E.-T.B.); shkim@jnu.ac.kr (S.-H.K.); gslee@jnu.ac.kr (G.L.); h.jeong@jnu.ac.kr (H.J.)

**Keywords:** time-of-flight, optical noise reduction filter, asynchronous integration time, 3D warping

## Abstract

A distance map captured using a time-of-flight (ToF) depth sensor has fundamental problems, such as ambiguous depth information in shiny or dark surfaces, optical noise, and mismatched boundaries. Severe depth errors exist in shiny and dark surfaces owing to excess reflection and excess absorption of light, respectively. Dealing with this problem has been a challenge due to the inherent hardware limitations of ToF, which measures the distance using the number of reflected photons. This study proposes a distance error correction method using three ToF sensors, set to different integration times to address the ambiguity in depth information. First, the three ToF depth sensors are installed horizontally at different integration times to capture distance maps at different integration times. Given the amplitude maps and error regions are estimated based on the amount of light, the estimated error regions are refined by exploiting the accurate depth information from the neighboring depth sensors that use different integration times. Moreover, we propose a new optical noise reduction filter that considers the distribution of the depth information biased toward one side. Experimental results verified that the proposed method overcomes the drawbacks of ToF cameras and provides enhanced distance maps.

## 1. Introduction

Time-of-flight (ToF) cameras produce a representation of the depth of a natural scene. They are used to reconstruct 3D models, track objects, and estimate obstacles since these depth sensors provide real-time three-dimensional depth maps. ToF sensors work by integrating a high-speed pulsed infrared light source with a conventional video camera. The depth sensor provides more robust depth information as compared to conventional depth estimation methods in homogenous regions, repetitive pattern regions, and occluded regions [1].

**Figure 1:**
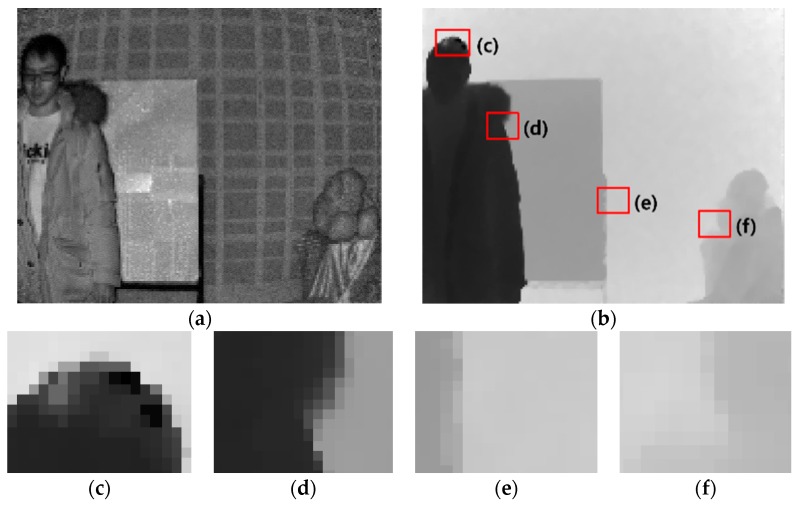
Problems in depth maps captured by time-of-flight (ToF) depth sensors. (**a**) Amplitude map, (**b**) distance image, (**c**) dark surface, (**d**) unmatched boundary, (**e**) shiny surface, and (**f**) unmatched boundary.

Nevertheless, these cameras still have some problems such as low resolution, inherent errors due to radiometric, geometric, and illumination variations [2,3]. Sergi Foix et al. presented five types of systematic errors; depth distortion errors, integration-time-related errors, built-in pixel-related errors, amplitude-related errors, and temperature-related errors [4]. Depth distortion errors occur when the infrared light is not emitted as theoretically planned (or generally sinusoidal). Integration-time-related errors occur when capturing the same scene at different integration times, causing different depth values in the entire scene. Built-in pixel-related errors arise from different material properties in CMOS gates and capacitor charge time delays during the signal correlation process. Amplitude-related errors are caused by low or overexposed reflected amplitudes. Temperature-related errors occur when the internal camera temperature affects depth processing. Light scattering errors [5], multipath errors [6], and object boundary ambiguity [7] have been identified as non-systematic errors. Compression may also result in distortion [8]. Figure 1 represents the problems in depth maps captured using ToF depth cameras.

Various solutions have been developed to enhance the depth maps captured by ToF depth sensors, most of which focus on the minimization of optical noise. Kim et al. exploited adaptive sampling, mesh triangulation, and Gaussian smoothing methods to enhance depth map information [9,10,11]. However, these methods do not suit real-time applications owing to the high complexity of the process. Moreover, the limitation of low resolution captured by ToF cameras is critical in computer vision applications. A hybrid camera system that combines a stereo camera and a ToF depth sensor was introduced to provide high-quality depth images [12,13,14]. Kopf et al. employed the concept of bilateral filters [15], which use color information to upsample a depth map [16]. Kim et al. presented a method to enhance depth maps by minimizing the unmatched boundary problem between depth and 2D image pairs using joint bilateral upsampling (JBU) and reduced the temporal depth flickering artefacts using a temporal filter [3]. Since raw depth sequences also suffer from depth flickering in a static region, the works of [17,18] used temporal filters to reduce the temporary fluctuation problem by simultaneously filtering several depth pairs to preserve depth consistency. 

The aim of this present study is to understand the errors caused by light reflection and absorption, and minimize them, thereby presenting a distance error correction method for ToF depth sensors. To solve the problem with ToF sensors, we exploited multi-ToF sensors and a novel filtering method. Section 2 describes the setup of the ToF depth sensor and its preprocessing. Section 3 gives the details of a novel weighted median filter method. Section 4 presents a distance error correction method for ToF depth sensors using different integration times. In Section 5, the proposed technique is validated using experiments. Finally, Section 6 concludes the paper.

## 2. ToF Camera Setup and Calibration

### 2.1. Light Reflection and Integration Time

**Figure 2:**
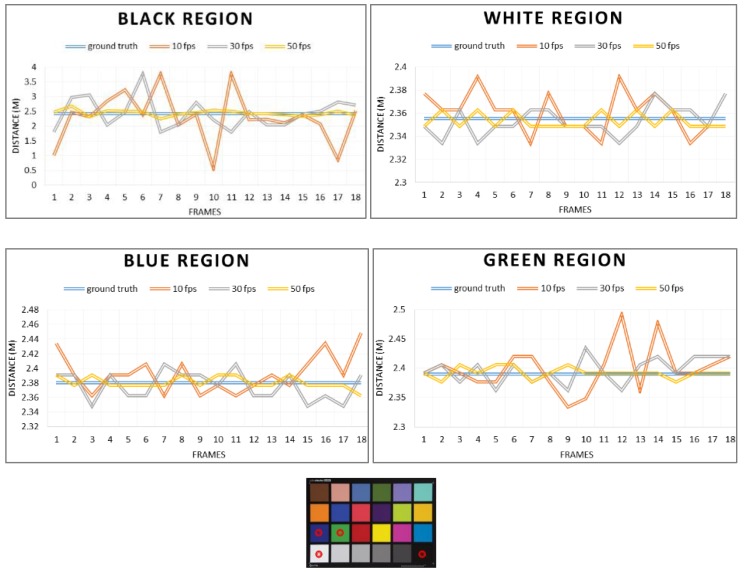
Depth information acquired in different colors at different integration times using the ToF depth camera.

We assumed that the source of the error in the ToF camera is the amount of light reflection and integration time. Therefore, the principle that the reflectance of light varies depending on the color was applied to this study. To verify that depth accuracy varies with color and time, we measured the depth using the color chart presented in Figure 2. ToF cameras acquire depth information with different accuracies for different colors. As we predicted, the ToF camera generates different depth information for different colors with different accuracy in Figure 2. The reason this phenomenon occurs is photons that are incident on pixels are captured and converted to electrons in the ToF camera. Since a black surface absorbs most of the photons while a white surface reflects most of the light, the depth accuracy of the black surface is very low compared to that of white or other colors. 

**Figure 3:**
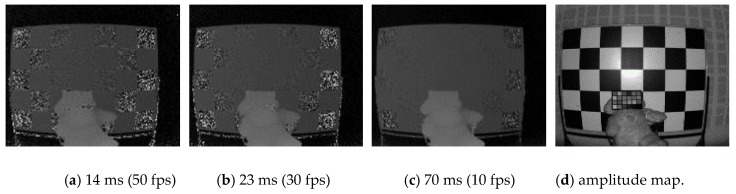
ToF depth error by integration time.

Experiments have been conducted to determine the factors in which integration time significantly affects error from ToF. Figure 3 represents the accuracy of depth, which increases with the integration time. Figure 3a–c represents distance maps captured at different integration times and Figure 3d shows the amplitude map. During a given integration time, the signals in the electrons collected for each pixel are proportional to the signal intensity. 

### 2.2. ToF Depth Sensor Setup

**Figure 4:**
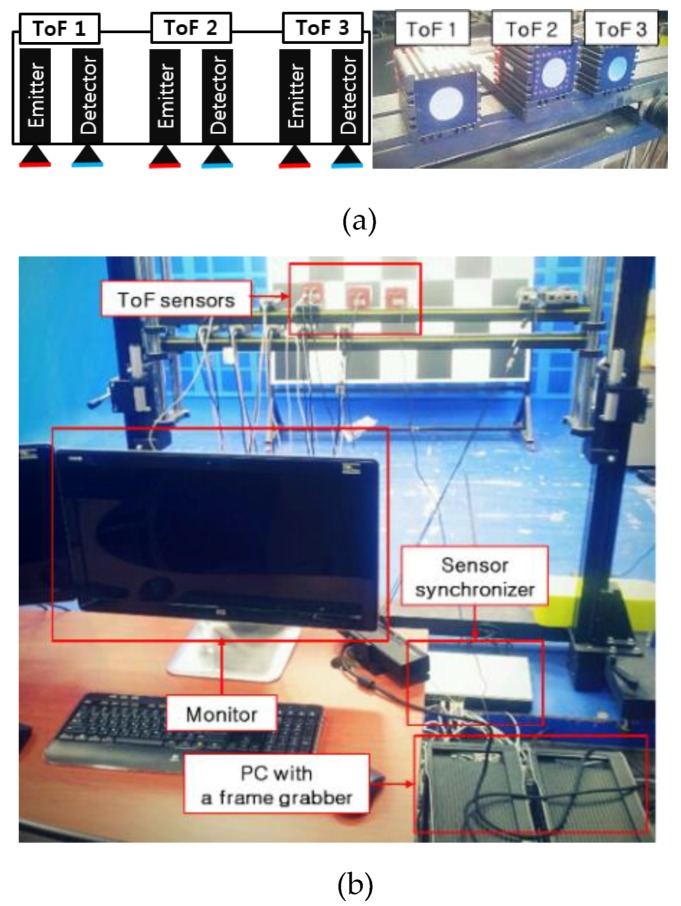
Depth sensor setup. (**a**) Position of the three ToF sensors and (**b**) operation example of ToF sensors.

Three ToF depth sensors were horizontally installed to share the depth information, as shown in Figure 4. The field of view (FOV) of the depth sensor should not be obstructed by any unwanted objects that could give rise to multiple path reflections. Therefore, ToF depth sensors should not be mounted on walls or tables far from the floor. The test apparatus to acquire and correct the ToF depth data was as illustrated in Figure 4b. Multiple ToF sensors are triggered by the ToF sensor synchronizer while acquiring depth information. The depth information is saved to the PC using a frame grabber, and the computer refines the ToF data.

**Figure 5:**
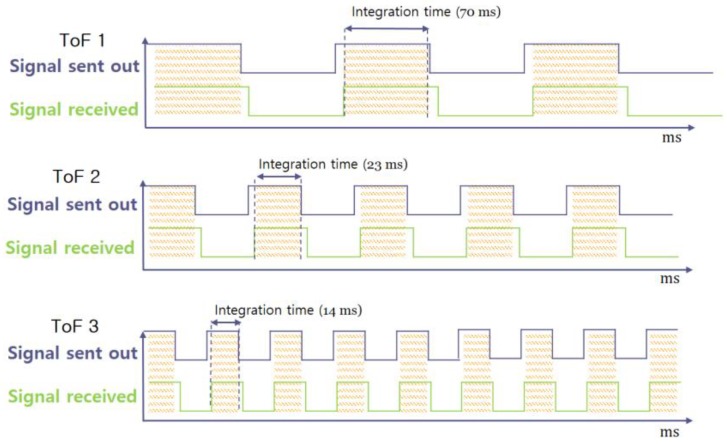
Three different signals with different integration times.

Typically, ToF cameras count the number of photons incident on pixels during an exposure time to estimate the distance. However, photons randomly fall onto the detector, making it very difficult to measure the average distance by counting photons. To overcome this problem, different integration times were set for each ToF depth sensor, i.e., approximately 70 ms (10 fps) for ToF 1, 23 ms (30 fps) for ToF 2, and 14 ms (50 fps) for ToF 3, as shown in Figure 5. Another drawback of the ToF camera is signal interference, which may cause significant errors. Infrared radiation (IR) does not exclusively transmit distance information for specific scene points. When multiple ToF depth sensors are operating in the same area, the illumination of the camera may interfere with other cameras. To avoid this interference, the ToF sensors were set at different frequencies (29, 30, and 31 MHz).

### 2.3. Camera Calibration for ToF Camera

ToF depth sensors follow the pinhole camera model that can be parametrized by intrinsic and extrinsic parameters. The camera parameters are the principal information of the camera for 3D image processing and application. The intrinsic parameters contain the camera’s internal features, such as the focal length, and they are represented as

(1) $$A=\left[\begin{array}{ccc} \alpha_{u} & s & u_{0}\\ 0 & \alpha_{v} & v_{0}\\ 0 & 0 & 1 \end{array}\right]$$

where α_u_ and α_v_ represent the focal lengths of the camera regarding pixel dimensions in u and v directions, respectively. u_0_ and v_0_ are the principal points where the principal axis intersects the image plane. s is the skew factor representing the non-orthogonality between the u and v axes. The extrinsic parameters entail the camera’s orientation and position information. In addition, the extrinsic parameters are composed of the rotation matrix, **R**, and translation vector, **t**. The camera projection matrix **P** is then composed of these parameters. The camera projection matrix is defined as

(2) P=A [R|t]

**Figure 6:**
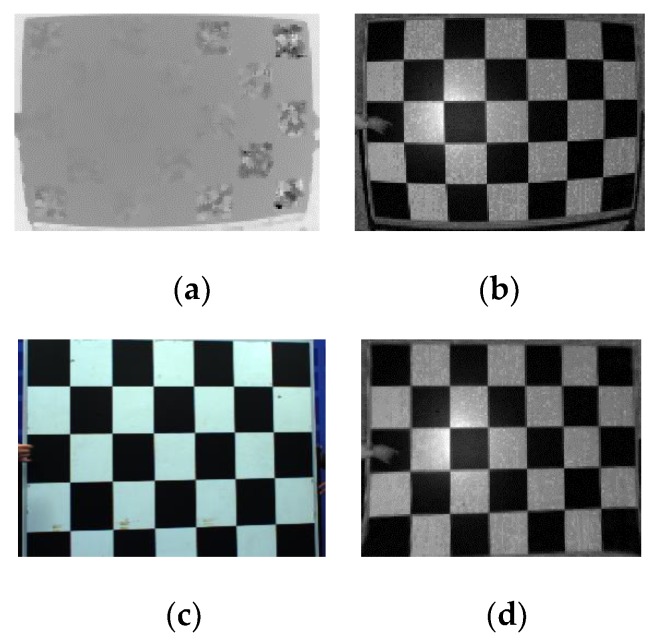
Depth map problems captured by ToF depth sensors. (**a**) Distance map, (**b**) amplitude map, (**c**) color image, and (**d**) corrected amplitude map.

The camera parameters are estimated by a camera calibration method using a chessboard [19]. However, errors occur during the camera calibration procedure, making it difficult to extract out real-world coordinates. To deal with this, we propose some methods to enhance ToF camera calibration. First, we exploit an amplitude map, instead of a distance map, to estimate camera calibration. The integration time is increased because the more the integration time, the higher the accuracy in amplitude measurement [2]. In addition, most ToF depth sensors have severe nonlinear lens distortion, especially the radial distortion shown in Figure 6b. The lens distortion is refined using a correction method, as shown in Figure 6d [4].

## 3. Optical Noise Reduction Filter

Inherent errors are generated in ToF depth sensors, which impact the results; it is therefore necessary to remove them before warping the accurate depth information from the neighboring ToF cameras to the center ToF camera. To reduce the artefacts, this section presents a new artefact removal filter. Most sets of distance values do not follow a normal distribution in practice. Data appear in the form of a distribution that is biased toward one side. Therefore, the skew-normal distribution is exploited as a weight for the weighted median filter. The proposed filter combines the original weighted median filter and modifies the weight kernel to suppress the noise while preserving the meaningful structure. In the following, we explain the proposed method in order.

### 3.1. Median Filter

Median filtering is a nonlinear method used to remove noise from images while maintaining edges [20]. This filter aims to replace the value of a pixel with the median of its neighbors. A median can be computed from a histogram, h(p,·), which calculates the population around the position p = (x, y).

(3) $$h\left(p,i\right)=\underset{x^{\prime }\in N\left(p\right)}{\sum }\delta \left(V\left(q\right)-i\right)$$

where V is a pixel value, N(p) is a window, q is the neighbor of p, i is the discrete bin index, and δ (·) is a Kronecker delta function. The median value can be easily chosen by accumulating this histogram.

### 3.2. Weighted Median Filter

A weighted median filter is a nonlinear digital filter with a window of length 2N + 1 [21]. The pixels are weighted in the local histograms. The weighted histogram is defined as

(4) $$\;h\left(p,i\right)=\underset{x^{\prime }\in N\left(p\right)}{\sum }w\left(p,q\right)\delta \left(V\left(q\right)-i\right)$$

where w = (w_-N_,…, w_0_,…, w_N_) is a weight vector that depends on an image, I, that can be different form V. Gaussian weights are generally assumed for weight w. In [15], bilateral weight was used to aggregate the stereo matching costs [22]. The median value is acquired by accumulating this histogram.

### 3.3. Skew Normal Distribution Weighted Median Filter

**Figure 7:**
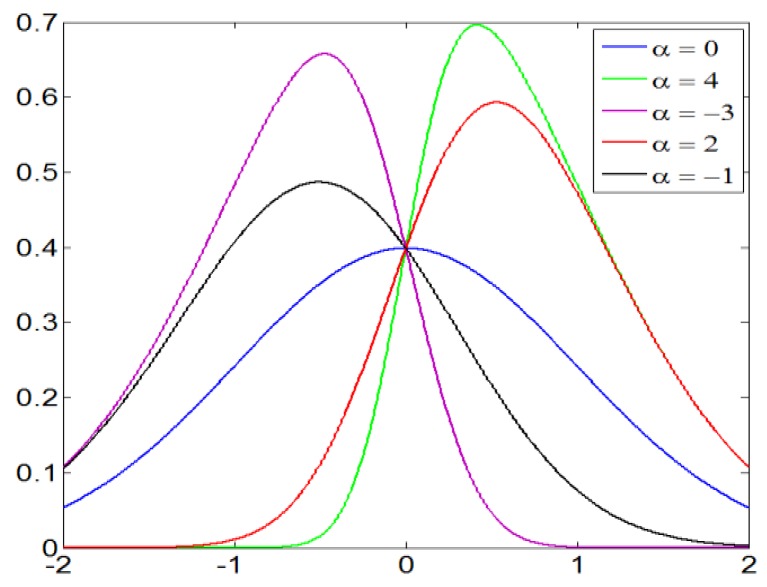
Skew normal distribution with parameter α.

The skew normal distribution is an extension of the normal (Gaussian) probability distribution to allow for non-zero skewness, as shown in Figure 7. The probability density function of skew normal distribution regarding parameter α is defined as

(5) f(x)=2∅(x)Φ(αx)

where ∅(x) denotes the standard normal probability density function given by

(6) $$\emptyset \left(x\right)=\frac{1}{\sqrt{2\pi }}e^{-\frac{x^{2}}{2}}$$

Here, Φ(x) is the cumulative distribution function represented as

(7) $$\Phi \left(x\right)=\int_{-\infty }^{x}\emptyset \left(t\right)\mathrm{dt}=\frac{1}{2}[1+\mathrm{erf}(\frac{x}{\sqrt{2}})]$$

where erf() is the error function. The error function is a special function, shaped like a sigmoid, that occurs in probability. The error function is defined as

(8) $$\mathrm{erf}\left(x\right)=\frac{2}{\sqrt{\pi }}\int_{0}^{z}e^{-t^{2}}\mathrm{dt}$$

A skew-normal distribution weighted histogram is defined as

(9) $$h\left(p,i\right)=\underset{x^{\prime }\in N\left(p\right)}{\sum }f\left(p,q\right)\delta \left(V\left(q\right)-i\right)$$

**Figure 8:**
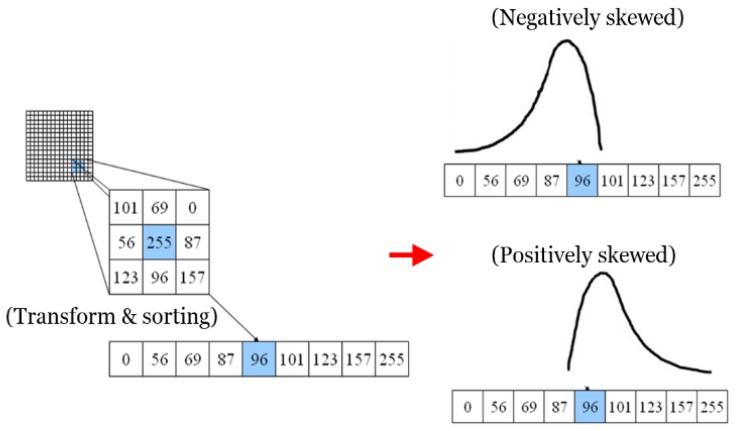
Example of skew-normal distribution weighted median filter.

Similar to the unweighted case, the median value is obtained by accumulating this histogram. Figure 8 shows an example of the proposed filter. Negative skew implies that the mass of the distribution is concentrated on the right, whereas positive skew implies that the mass of the distribution is concentrated on the left.

The weights are changed depending on α. Examples of positively and negatively skewed distributions are illustrated in Figure 8. The equation for estimating α is defined as

(10) $$\alpha =\{\begin{array}{c} S_{P},\;\mathrm{if}\;\left|\mathrm{med}-\min\right|>\left|\mathrm{med}-\max\right|\\ S_{N},\;\mathrm{if}\;\left|\mathrm{med}-\min\right|<\left|\mathrm{med}-\max\right|\\ 0,\;\mathrm{otherwise}\; \end{array}$$

where S_p_ and S_N_ are the weights for positively and negatively skewed distributions, respectively, and med is the median of a vector, min and max are the minimum and maximum values of a vector. Figure 8 shows an example of skew-normal distribution weighted median filter. Given the window, we first transform a 2D matrix to a 1D array and sort it. After obtaining α, the 1D array is filtered. 

## 4. Distance Error Correction Using Different Integration Time

In this section, we demonstrate the ambiguity in depth information in shiny and dark surfaces. We present a method to detect the shiny and dark surfaces and correct the regions. 

**Figure 9:**
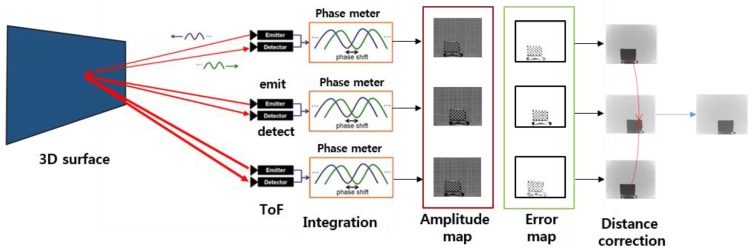
Operation of the proposed distance error correction method.

Figure 9 schematically illustrates the operation of the proposed distance error correction method using multiple ToF information captured at different integration times. Each ToF sensor integrates light after emitting it. Amplitude maps were exploited to estimate the error in each ToF data. Given the detected regions, accurate depth information is warped to correct the distance.

### 4.1. Shiny and Dark Surface Detection

**Figure 10:**
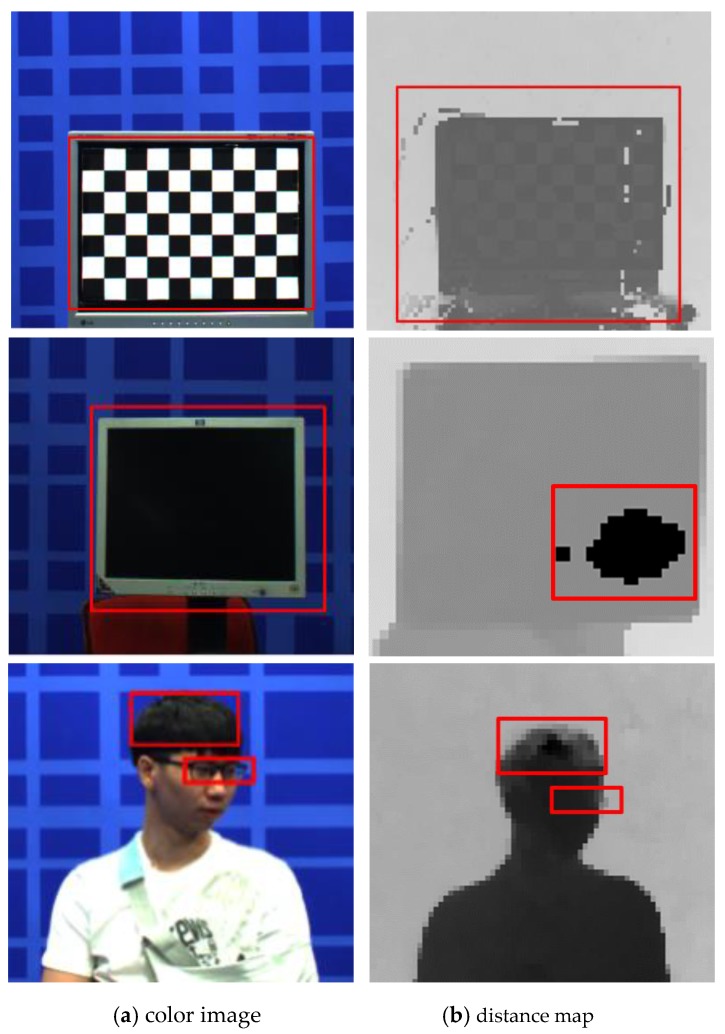
Examples of shiny and dark surfaces. First row represents chessboard pattern, second row shows a monitor, and third row shows hair and glasses.

Shiny surfaces are poor absorbers and good reflectors of radiation. Typically, shiny surfaces reflect several light waves. In contrast, dark and matte surfaces are good absorbers and poor reflectors of radiation. Figure 10 presents pictures of a chessboard pattern, hair, glasses, and monitor. Since shiny and dark surfaces create large amounts of noise for ToF depth sensors, a distance map is strongly affected by these surfaces. Therefore, the problem of shiny and dark surfaces should be solved.

ToF depth sensors provide an amplitude map that represents the height of the sine wave or the brightness of the light. Too low an amplitude surface gives a dark and matte surface, while too high gives a shiny surface. Therefore, the amplitude map I_A_ is exploited to detect error regions. The error detection function, R_e_(), is defined as

(11) $$R_{e}\left(x,y\right)=\{\begin{array}{c} E_{d},{\;\mathrm{if}\;I}_{A}\left(x,y\right)<T_{D}\\ E_{s},{\;\mathrm{if}\;I}_{A}\left(x,y\right)>T_{S}\\ E_{n},\;\;\;\;\;\;\mathrm{otherwise}\; \end{array}$$

where T_D_ and T_S_ are the thresholds of dark and shiny surfaces, respectively. E_d_ is the error pixel for the dark surface, E_s_ is the error pixel for the shiny surface, and E_n_ denotes no error. Using Equation (11), each amplitude map is calculated to detect the error regions.

### 4.2. Error Correction

**Figure 11:**
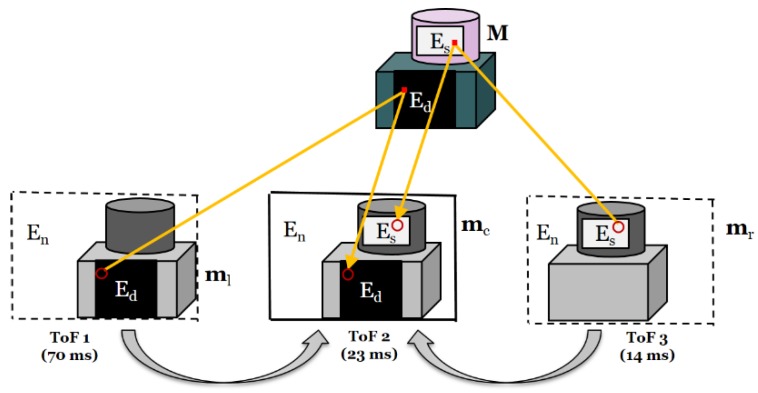
Error correction using 3D warping.

The disparity map obtained from ToF sensors after longer integration usually ensures higher accuracy in distance measurement in dark and matte regions while the disparity maps from ToF by short integration time usually ensures a higher accuracy of distance measurement in shiny surfaces. In this study, the disparity map of ToF 1 represented accurate information for the shiny surfaces and the disparity map of ToF 3 represented accurate information for the dark and matte surfaces. Given the detected regions, accurate depth information was warped from ToF 1 and ToF 3 to ToF 2. As illustrated in Figure 11, the 3D image warping process consists of two steps, i.e., backward projection with depth data and forward projection. Let m_l_ and m_c_ be the corresponding pixels in the viewpoint images of ToF 1 and ToF 2 in the set of the error pixels E_d_, respectively. Based on the pin-hole camera model, the pixel point m_l_ can be defined by the camera parameters as

(12) $$m_{l}=A_{l}\cdot R_{l}\cdot M+A_{l}\cdot t_{l}$$

The next step is finding the corresponding pixel position m_c_ from the viewpoint of ToF 2. The point in the world coordinate M is projected onto the viewpoint of ToF 2 using its camera parameters as

(13) $$\begin{array}{cc} {\;m}_{c} & =A_{c}\cdot R_{c}\cdot M+A_{c}\cdot t_{c}\\ & =A_{c}\cdot R_{c}\cdot {\;R}_{l}^{-1}\cdot A_{l}^{-1}\cdot m_{l}-A_{c}\cdot R_{c}\cdot {\;R}_{l}^{-1}\cdot t_{l}+A_{c}\cdot t_{l} \end{array}$$

Similar to the case of ToF 1, the points in ToF 3 can be warped in the set of error pixels, E_s_.

## 5. Experimental Results

**Figure 12:**
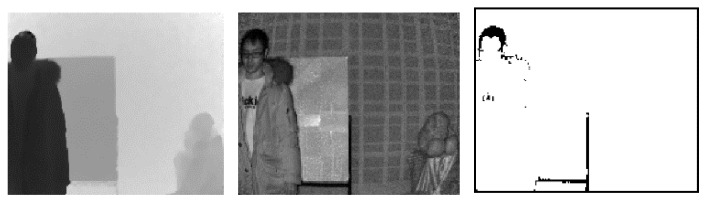

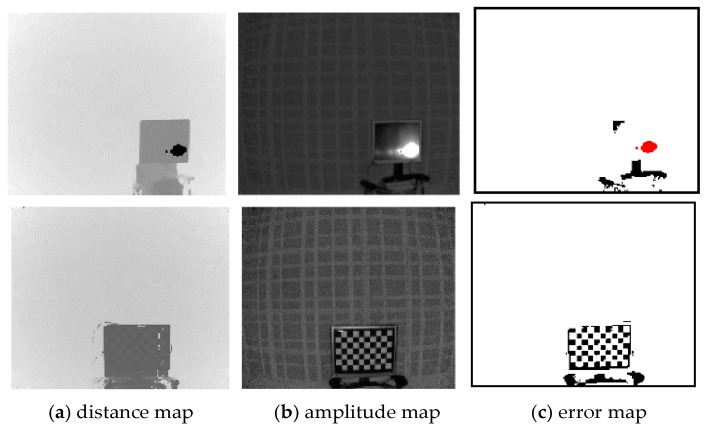
Results of error detection.

First, for the qualitative evaluation of the ToF depth sensor, the test dataset was captured by three ToF depth sensors. Figure 12 illustrates the results of error detection using the amplitude map. Figure 12a shows that errors that occur in the shiny and dark regions. As shown in Figure 12c, the red region indicates the shiny surface E_s_ and the black region denotes the dark and matte surface E_d_. We confirm that the error region is accurately determined through the error detection method, as the result of Figure 12c.

**Figure 13:**
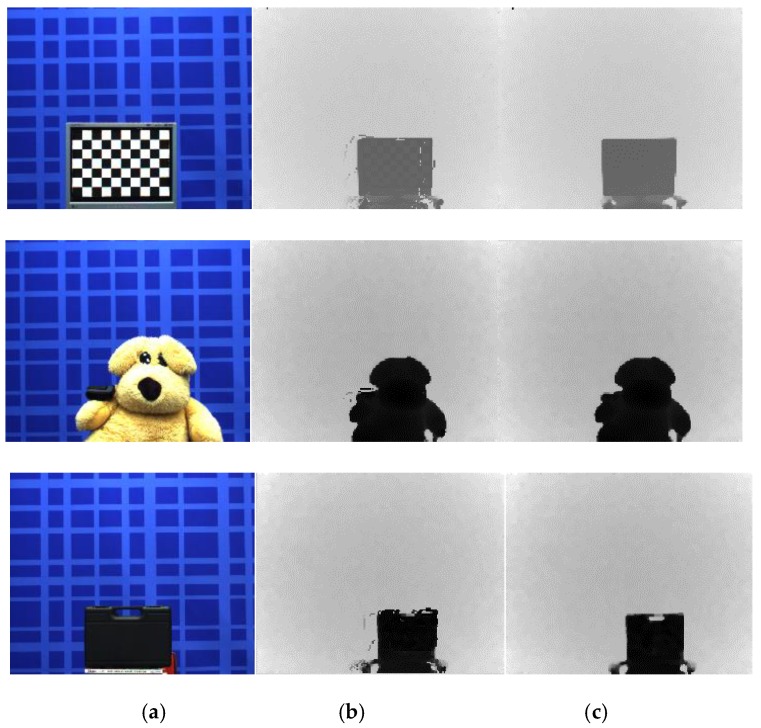
Results of the error correction method using different integration times. (**a**) Color image, (**b**) distance map (ToF 2), and (**c**) corrected distance map.

As shown in Figure 13, real scenes captured by the ToF depth sensors are exploited to verify the proposed error correction method using three ToF depth sensors subjectively. In Figure 13b, visual artefacts are observed in shiny and dark surfaces. Figure 13c presents the noises in depth map captured by ToF 2, recovered using the proposed error correction method. Also, errors around the objects are remarkably reduced in Figure 13c.

**Figure 14:**
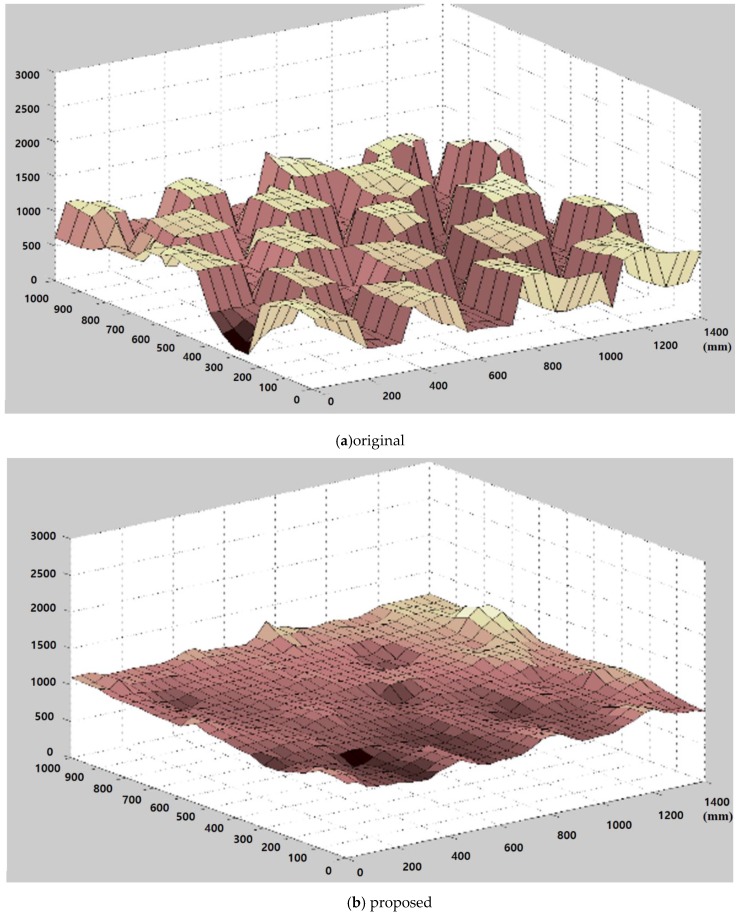
Three-dimensional surface plot using zoomed-in depth.

**Figure 15:**
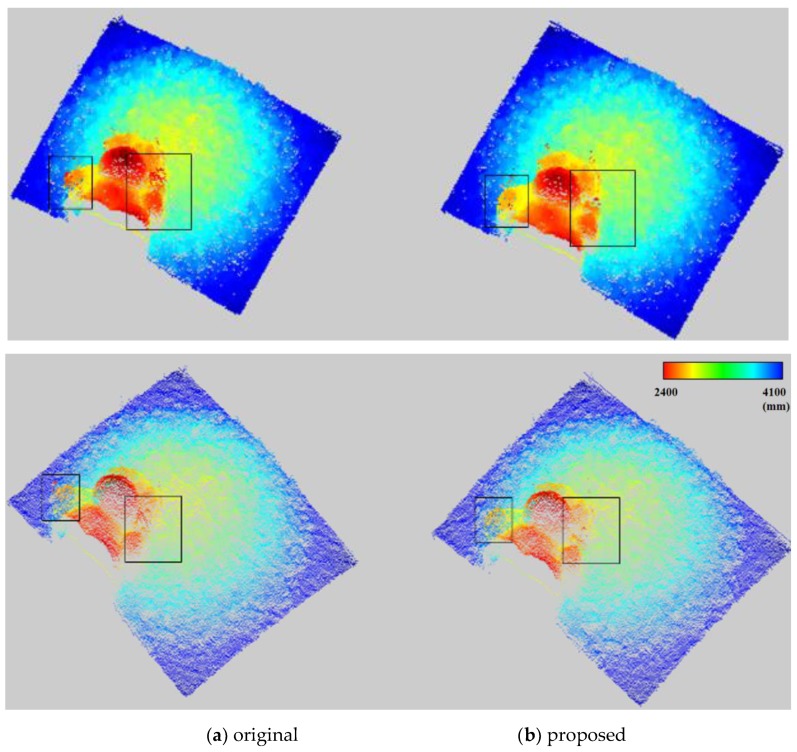
Three-dimensional point cloud.

Figure 14 illustrates the 3D graph of the distance map using the close-up depth map from Figure 13. Figure 14a shows a fluctuating 3D surface. However, Figure 14b shows that the fluctuations in the 3D surface are suppressed dramatically. Moreover, to precisely evaluate the performance of the algorithm, the original distance map and the result from the proposed method are converted to a 3D point cloud, as shown in Figure 15, and the rectangles are drawn. The points of the 3D point cloud are scattered in the rectangles in Figure 15a. However, Figure 15b shows the refined original distance map by the proposed method in the error regions.

**Figure 16:**
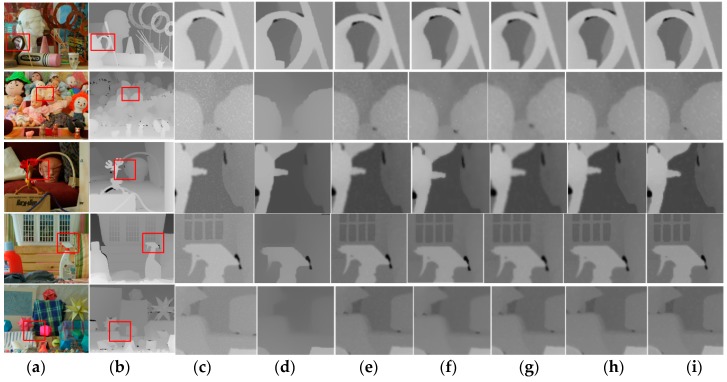
Results and comparison. (**a**) Original color images, (**b**) original depth map, (**c**) synthesized noisy images, (**d**) RTV [24], (**e**) bilateral filter [15], (**f**) domain transform filter [25], (**g**) mean filter, (**h**) median filter, and (**i**) SWMF (proposed).

Figure 16 presents the results of the proposed artefact filtering method and conventional methods. In the case of relative total variation (RTV) and domain transform filter, even though meaningful edges were preserved, they generate inaccurate depth information in homogenous areas. As shown in Figure 16e, the bilateral filter blurs the depth information on object boundaries more compared to other methods. In contrast, the proposed method better suppresses the Gaussian noise while preserving major edges, and sharpens the edges, in comparison to the conventional methods.

For the quantitative evaluation of the performance of the propoased skew-normal distribution weighted median filter(SWMF), test images from the Middlebury database [23] were used for the ground truth of the synthesized noisy images, to which Gaussian noise was artificially added, and the contours were blurred. The parameters for SWMF were fixed as follows; S_P_ = 2 or S_N_ = −2, and the window size was 3 in Equation (10). The root mean squared error (RMSE) was used to quantitatively analyze the proposed method, which is the square root of the average of the square of all errors. The RMSE is used as a widespread in the field of computer vision because it makes for an excellent general-purpose error metric for numerical predictions. The results of performance comparison are presented in Table 1. Based on this, the proposed filter outperforms conventional methods based on RMSE. Error in the three-dimensional information on a particular application, such as 3D reconstruction or intermediate view synthesis, is very significantly involved in the performance. Even though the RMSEs have increased by 10%, this result is very significant at the application level.

**Figure 17:**
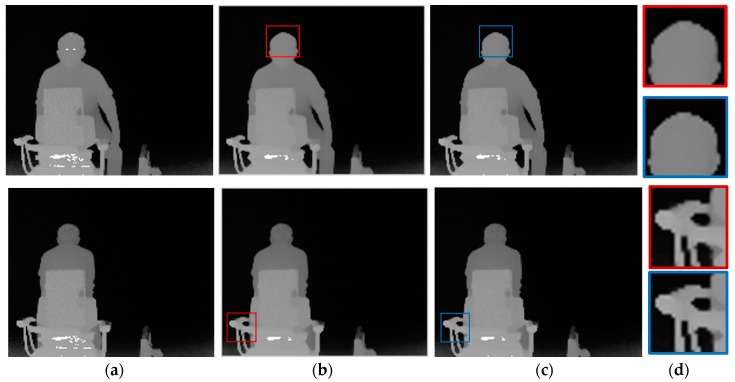
Experimental results with real-world data. (**a**) Original, (**b**) median filter, (**c**) SWMF (proposed), and (**d**) closed-up images.

We also exploited real-world examples to test the proposed skew-normal distribution weighted median filter. Figure 17 represents the filtered distance maps of the proposed method and the median filter method. The optical noise in the distance map was reduced using the proposed method while efficiently preserving the meaningful edges without any unintended visual artefacts in Figure 17c, as compared to the conventional method. Figure 17d, with enlarged depth maps, demonstrates that the proposed method outperforms the previous method in the contour regions.

**Figure 18:**
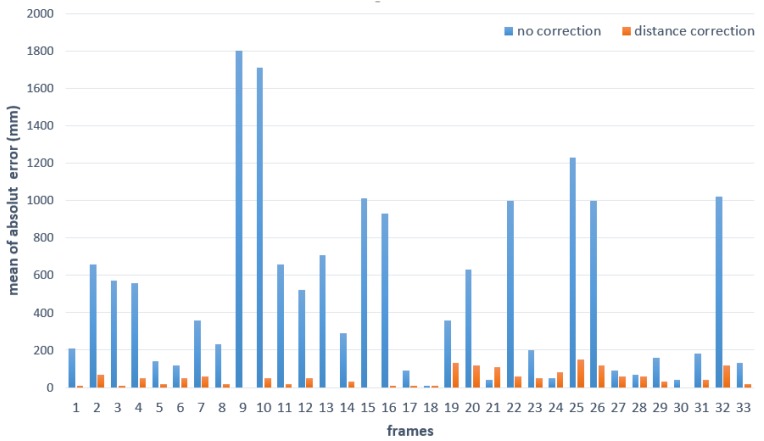
Distance error with and without correction.

In addition, we performed a quantitative evaluation of the proposed method. Because it is hard to obtain the actual distance, we used a square plane to generate ground truth. The reference plane was 50 × 50 mm in size and positioned at a distance of 1000 mm from the camera. The distance between the plane and the ToF camera was measured and compared with the ground truth. We measured the distance error to evaluate the quality of the proposed method, and then compared it with the quality of the raw data. Figure 18 shows the distance error for each sequence. It shows that the proposed method reduces the error dramatically.

## 6. Conclusions

This study proposes a distance error correction method for ToF depth sensors using different integration times and a novel weighted median filter. The proposed SWMF outperformed the conventional approaches and efficiently reduced the artefacts while preserving the important structure. Moreover, by using three ToF depth sensors, the proposed method removed the inherent crucial depth errors in shiny and dark surfaces, thereby generating an accurate distance map. In addition, the cost and size of ToF cameras have been reduced in recent years. Therefore, even if three ToF cameras are used, there is little burden in size and cost. The advantage of predicting high-quality depth information can complement price and size. Consequently, the proposed distance error correction method can be potentially implemented in various 3D applications. Future work should aim to include inter-frame information to enforce time consistency and further increase the accuracy of distance information.

## Figures and Tables

**Table 1:** Root-mean-square-error (RMSE) comparison.

	Art	Dolls	Laundry	Moebius	Reindeer
Synthesized noisy image	3.304	4.542	2.534	2.667	2.682
RTV [24]	4.832	4.789	3.209	3.703	3.496
Bilateral filter [17]	3.495	4.544	2.642	2.750	2.802
Domain transform [25]	3.292	4.320	2.544	2.655	2.621
Mean filter	3.651	4.578	2.754	2.829	2.894
Median filter	2.446	2.490	1.573	1.704	1.735
SWMF (proposed)	1.993	2.451	1.342	1.664	1.585

## References

[1] Scharstein D., Szeliski R. (2002). A taxonomy and evaluation of dense two-frame stereo correspondence algorithms. Int. J. Comput. Vision.

[2] Hansard M., Lee S., Choi O., Horaud R.P. (2012). Time-of-flight Cameras: Principles, Methods and Applications.

[3] Kim S., Cho J., Koschan A., Abidi M. Spatial and temporal enhancement of depth images captured by a time-of-flight depth sensor. Proceedings of the 20th International Conference on Pattern Recognition.

[4] Foix S., Alenya G., Torras C. (2011). Lock-in time-of-flight (ToF) cameras: a survey. IEEE Sens. J..

[5] Mure-Dubois J., Hügli H. Real-Time Scattering Compensation for Time-of-Flight Camera. http://biecoll.ub.uni-bielefeld.de/index.php/icvs/article/view/240.

[6] Fuchs S. Multipath interference compensation in time-of-flight camera images. Proceedings of the 20th International Conference on Pattern Recognition.

[7] Reynolds M., Doboš J., Peel L., Weyrich T., Brostow G.J. Capturing time-of-flight data with confidence. Proceedings of the IEEE Computer Vision and Pattern Recognition.

[8] Agresti G., Minto L., Marin G., Zanuttigh P. (2019). Stereo and ToF data fusion by learning from synthetic data. Inf. Fusion.

[9] Wang A., Qiu T., Shao L. (2009). A simple method of radial distortion correction with centre of distortion estimation. J. Math. Imaging Vision.

[10] Song Y., Ho Y. (2017). High-resolution depth map generator for 3D video applications using time-of-flight cameras. IEEE Trans. Consum. Electron.

[11] Kim S., Cha J., Ryu J., Lee K. (2006). Depth video enhancement of haptic interaction using a smooth surface reconstruction. IEICE Trans. Inf. Syst..

[12] Zhu J., Wang L., Yang R., Davis J. Fusion of time-of-flight depth and stereo for high accuracy depth maps. Proceedings of the 2008 IEEE Conference on Computer Vision and Pattern Recognition.

[13] LEE J., PARK H. (2015). Efficient synthesis-based depth map coding in AVC-compatible 3D video coding. IEEE Trans. Circuits Syst. Video Technol..

[14] Ho Y., Lee E., Lee C. Multiview Video Test Sequence and Camera Parameters. https://www.researchgate.net/publication/259756600_Contribution_Poznan_Multiview_Video_Test_Sequences_and_Camera_Parameters.

[15] Tomasi C., Manduchi R. Bilateral filtering for gray and color images. Proceedings of the Sixth International Conference on Computer Vision.

[16] Kopf J., Cohen M.F., Lischinski D., Uyttendaele M. (2007). Joint bilateral upsampling. ACM Trans. Graph..

[17] Matyunin S., Vatolin D., Berdnikov Y., Smirnov M. Temporal Filtering for Depth Maps generated by Kinect Depth Camera. Proceedings of the 3DTV Conference: The True Vision—Capture, Transmission and Display of 3D Video (3DTV-CON).

[18] Garcia F., Aouada D., Mirbach B., Ottersten B. Spatio-temporal ToF data enhancement by fusion. Proceedings of the 19th IEEE International Conference on Image Processing.

[19] Zhang Z. (2000). A flexible new technique for camera calibration. IEEE Trans. Pattern Anal. Mach. Intell..

[20] Huang T., Yang G., Tang G. (1979). A fast two-dimensional median filtering algorithm. IEEE Trans. Acoust. Speech Signal Process..

[21] Yin L., Yang R., Gabbouj M., Neuvo Y. (1996). Weighted median filters: a tutorial. IEEE Trans. Circuits Syst. II: Analog Digital Signal Process.

[22] Ma Z., He K., Wei Y., Sun J., Wu E. Constant Time Weighted Median Filtering for Stereo Matching and beyond. https://www.cv-foundation.org/openaccess/content_iccv_2013/html/Ma_Constant_Time_Weighted_2013_ICCV_paper.html.

[23] Middlebury Stereo Vision Page. http://vision.middlebury.edu/stereo/.

[24] Xu L., Yan Q., Xia Y., Jia J. (2012). Structure extraction from texture via relative total variation. ACM Trans. Graph..

[25] Gastal E.S., Oliveira M.M. (2011). Domain Transform for Edge-Aware Image and Video Processing. ACM Trans. Graph..

